# A Rare Case of Triple Positive Metachronous Breast Cancer

**DOI:** 10.1177/2324709619892106

**Published:** 2019-12-20

**Authors:** Hardik S. Chhatrala, John Khuu, Lara Zuberi

**Affiliations:** 1 University of Florida Health at Jacksonville, Jacksonville, FL, USA

**Keywords:** breast cancer, brain metastasis, metachronous, HER-2 positive, triple positive, pathologic complete remission

## Abstract

Metachronous contralateral breast cancer (MCBC) is defined as contralateral breast cancer (BC) diagnosed more than 1 year after previous BC diagnosis. More BC survivors are at risk of MCBC given improved life expectancy with the availability of advanced cancer care. Estrogen receptor/progesterone receptor negative and HER-2-positive status of first BC are independent risk factors for the development of MCBC. We present a rare case of triple positive (estrogen receptor, progesterone receptor, HER-2 positive) MCBC patient who eventually developed brain metastasis within 15 months despite a near complete pathologic response of primary tumor. This case highlights that even in this era of antiestrogen and anti-HER-2 therapies, triple positive MCBC can have an aggressive clinical course, especially with brain metastasis as the first sign of metastasis.

## Introduction

Triple positive breast cancer (BC; estrogen receptor/progesterone receptor [ER/PR]/HER-2 positive) constitutes only about 10% of all BCs.^1^ Metachronous contralateral BC (MCBC) is defined as contralateral BC diagnosed more than 1 year after previous BC diagnosis. MCBC is not common and the incidence has been reported between 1.4% and 11.8%.^2^ Hormonal receptor (HR)-negative first BC has a higher incidence of contralateral BC compared with HR-positive first BC.^2-4^ Human epidermal growth factor receptor 2 (HER-2)-positive first BC has a higher incidence of contralateral BC compared with HER-2-negative first BC.^2,3^ However, there are very few reported cases of contralateral metachronous triple positive BC.

## Case Description

**Figure 1.**
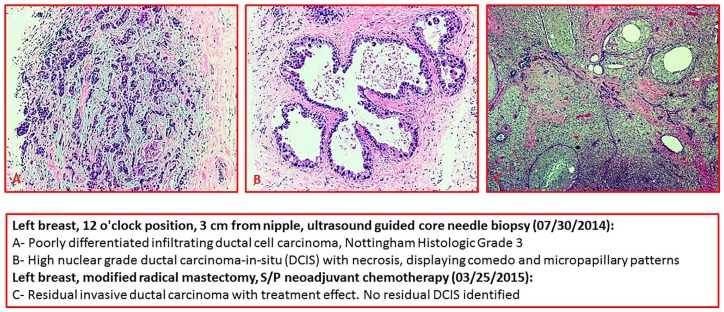
Left breast, 12 o’clock position, 3 cm from nipple, ultrasound-guided core needle biopsy (July 30, 2014): (A) Poorly differentiated infiltrating ductal cell carcinoma, Nottingham Histologic Grade 3. (B) High nuclear grade ductal carcinoma in situ (DCIS) with necrosis, displaying comedo and micropapillary patterns. Left breast, modified radical mastectomy, S/P neoadjuvant chemotherapy (March 25, 2015): (C) Residual invasive ductal carcinoma with treatment effect. No residual DCIS identified.

A 33-year-old Caucasian female presented with stage IIIB (T4N1M0) poorly differentiated (grade 3) invasive ductal carcinoma of the right breast, which was positive for ER, PR, and HER-2. She was treated with neoadjuvant TCHP (docetaxel, cyclophosphamide, trastuzumab, and pertuzumab) with good clinical response, and she underwent a modified radical mastectomy with level II axillary lymph node dissection, showing partial pathological response ypT2N0M0 (Figure 1). This was followed by adjuvant radiation, and 1 year of adjuvant herceptin. She declined ovarian suppression, and she was started on adjuvant tamoxifen immediately following healing from surgery. Genetic testing was negative for BRCA1 and 2, but was positive for a variant of the BARD mutation, which has unclear clinical significance.^5,6^

**Figure 2.**
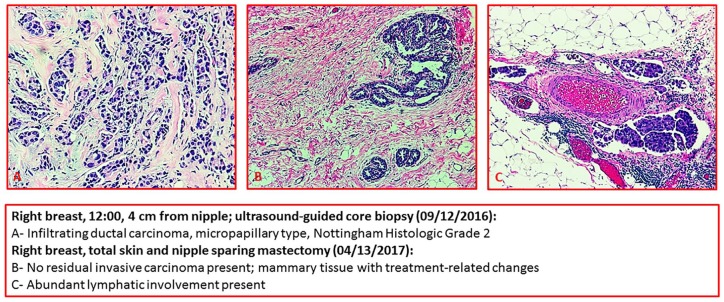
Right breast, 12:00, 4 cm from nipple; ultrasound-guided core biopsy (September 12, 2010): (A) Infiltrating ductal carcinoma, micropapillary type, Nottingham Histologic Grade 2. Right breast, total skin and nipple-sparing mastectomy (April 13, 2017): (B) No residual invasive carcinoma present; mammary tissue with treatment-related charges. (C) Abundant lymphatic involvement present.

Two years after her initial diagnosis, she presented with a fast-growing firm lump in the contralateral left breast measuring 6 cm × 6 cm. Biopsy confirmed invasive ductal carcinoma grade 2, ER+/PR+/HER2+ breast cancer (Figure 2). Metastatic workup was negative other than nonspecific areas in the ribs, biopsy of which revealed reactive cells. She was managed with neoadjuvant TCHP followed by surgical resection, radiation, planned 1 year adjuvant trastuzumab and pertuzumab followed by a different anti-endocrine regimen, with ovarian suppression and aromatase inhibitor. She underwent mastectomy, showing a complete pathological response in the primary tumor, but with residual isolated tumor cells in 1 out of 4 axillary nodes and lymphovascular invasion, ypTx N0(itc)(sn; Figure 2).

While on adjuvant trastuzumab, petuzumab, and luprolide, she developed multiple brain parenchymal and leptomeningeal metastases within 15 months of initial tumor diagnosis (Figure 2). This led to multiple neurological complications including seizure, hydrocephalus, brain stem compromise, and eventual coma. Due to poor prognosis, family elected no further treatment and the patient was transitioned to hospice care.

## Discussion

Women with history of BC have 1.6 times higher risk of developing MCBC than women who develop their first BC.^3^ Population-based studies have consistently shown ER/PR negative first BC have higher risk of developing MCBC compared with ER/PR positive first BC patients.^2-4,7^ This risk ranges from 1.4 to 10 times based on the study.^3,7^ The availability of antiestrogen therapies that is given in adjuvant setting to prevent BC recurrence on the same or contralateral side in HR-positive patients explains some of this difference in risk.^8,9^ HER-2 positivity refers to HER-2 gene overexpression in breast tumor and is uncommon, comprising about 15% to 20% of all primary BCs.^10^ HER-2 positivity increases the risk for developing contralateral BC by 5.9 times compared with HER-2-negative BC patients.^11^ However, with the introduction of targeted anti-HER-2 therapies like trastuzumab and pertuzumab, we are seeing better overall survival for this subgroup of BC patients.^12^ In patients with “triple positive” (ER, PR, and HER-2-positive) BCs, it not clearly understood whether ER/PR receptor or HER-2 receptor is a stronger driver for carcinogenesis and whether any synergy exists between them.

Pathologic complete response with neoadjuvant chemotherapy correlates with significantly improved event-free survival and overall survival in patients with localized BC.^13,14^ Our case is unique in the sense that the contralateral primary tumor achieved a near complete pathologic complete response, yet it had an aggressive course leading to brain metastases. The introduction of antiestrogen and anti-HER-2 therapies has revolutionized BC management. However, approximately 10.8% to 14.3% HER-2-positive early BC patients develop brain metastasis with an overall survival of less than 2 years.^15^ Among factors elucidated by Maurer et al,^15^ our patient had tumor size >2 cm and age <40 years as risk factors for brain metastasis in early HER-2-positive BC patients. It was very unusual to detect relatively rapidly fatal triple positive metachronous BC of the contralateral breast in the era of neoadjuvant dual antiestrogen and anti-HER-2-targeted therapies despite response to primary tumor.

There are no clear guidelines specific to this unusual circumstance. Our patient was treated with standard chemotherapy with TCHP. Because she was previously exposed to trastuzumab and pertuzumab, one has to consider the potential for cardiotoxicity with additional cycles of the same. However, the safety data are available for this only from the metastatic setting, where patients are on these treatments long term. Given the rarity of triple positive breast cancer and the rarity of this occurring in both breasts at different time points lends credence to the need for further research in the area of triple positive metachronous BC. This is especially relevant in a young patient who elected not to undergo prophylactic mastectomy and also to further look into the relevance of the BARD mutation and the risk of brain metastasis in this setting.
